# Lessons Learned From the Battlefield and Applicability to Veterinary Medicine – Part 2: Transfusion Advances

**DOI:** 10.3389/fvets.2021.571370

**Published:** 2021-05-07

**Authors:** Thomas H. Edwards, Anthony E. Pusateri, Erin Long Mays, James A. Bynum, Andrew P. Cap

**Affiliations:** ^1^U.S. Army Institute of Surgical Research, Joint Base San Antonio, San Antonio, TX, United States; ^2^Veterinary Specialty Services, Manchester, MO, United States

**Keywords:** transfusion, chilled whole blood, freeze dried plasma, chilled platelets, dog, trauma, hemostatic resuscitation

## Abstract

Since the inception of recent conflicts in Afghanistan and Iraq, transfusion practices in human military medicine have advanced considerably. Today, US military physicians recognize the need to replace the functionality of lost blood in traumatic hemorrhagic shock and whole blood is now the trauma resuscitation product of choice on the battlefield. Building on wartime experiences, military medicine is now one of the country's strongest advocates for the principle of hemostatic resuscitation using whole blood or balanced blood components as the primary means of resuscitation as early as possibly following severe trauma. Based on strong evidence to support this practice in human combat casualties and in civilian trauma care, military veterinarians strive to practice similar hemostatic resuscitation for injured Military Working Dogs. To this end, canine whole blood has become increasingly available in forward environments, and non-traditional storage options for canine blood and blood components are being explored for use in canine trauma. Blood products with improved shelf-life and ease of use are not only useful for military applications, but may also enable civilian general and specialty practices to more easily incorporate hemostatic resuscitation approaches to canine trauma care.

## Introduction

This review addresses resuscitation lessons learned in the Afghanistan and Iraq conflicts and describes how those can be translated to canine trauma resuscitation. Over the past 20 years, advances in trauma resuscitation have been driven by the simultaneous goals of optimizing patient outcomes while reducing logistical requirements. This resulted in guidelines and new development initiatives that address both challenges. This article will discuss how new transfusion approaches may apply to canine medicine, both on the battlefield and in the setting of civilian general or specialty practice. The focus will primarily be placed on the importance of resuscitating patients with hemorrhagic shock as soon as possible following injury using blood products, with emphasis on new approaches for accomplishing this in a logistically feasible manner.

## The Historical Case for Hemostatic Resuscitation

Blood transfusions have been used in human military medicine for over 100 years (1). During World War I, whole blood was the primary resuscitation product for US combat casualties on the battlefield (2). During World War II, freeze-dried plasma (FDP) was primarily used for combat trauma resuscitation due to its stability at ambient temperature and ease of transport into and around the battlefield; although whole blood was provided during surgery when available. Due to hepatitis transmission associated with the freeze-dried pooled plasma, its use was largely abandoned after WWII (3). Whole blood transfusion remained the optimal resuscitation product until the Viet Nam era, when crystalloids became the primary resuscitative fluid for service members injured on the battlefield (4). This shift was due to the ongoing risk of transfusion-transmitted infection and an overemphasis of research suggesting that the interstitial space needed resuscitation for trauma with 1-2 L (or 2-3 times the volume of blood shed) of crystalloids, only after which blood products should be given (5). This led to resuscitation with large volumes of crystalloids, with associated sequelae such as acute respiratory distress syndrome (coined “Da Nang Lung”), compartment syndrome, and multi-organ dysfunction (6). During this same era, civilian blood banks transitioned to an inventory of primarily blood components rather than whole blood, which facilitated targeted treatment of specific hematologic disorders and aided in donor screening for hepatitis and other infectious diseases (7). Therefore, in the last decades of the twentieth century, “blood” to treat hemorrhage was administered almost exclusively as component parts and often not in balanced proportions.

## Hemostatic Resuscitation Refined

In the past 20 years, it has become clear that aggressive resuscitation with crystalloids or synthetic colloids is related to coagulopathy and increased bleeding, and a shift toward earlier blood-based (or “hemostatic”) resuscitation has taken place in both civilian and military human trauma care (8). Initially, packed red blood cells (pRBC) were the predominant product used in this blood-based resuscitation, but over time, it became apparent that adding plasma and platelets improved outcomes (9–12). A large retrospective study of military casualties over a 10-year period revealed that patients initially transfused with higher ratios of plasma and platelet products had a decreased mortality (13). Military practice shifted toward providing blood products to achieve a 1:1:1 ratio [plasma:platelets:red cells (1 unit of plasma:1 six pack of apheresis platelets:1 unit of pRBCs)], when possible (14). Following the publication of a prospective randomized clinical trial which supported the advantage of a balanced ratio of fresh frozen plasma, platelets, and pRBC to reduce death by exsanguination, this practice became increasingly accepted in civilian centers (15).

While it has become clear that blood products are optimal for treatment of human patients with hemorrhagic shock and severe trauma, it appears that the *timing* of blood product administration can further enhance the benefit of this strategy. After reaching a trauma center, the benefits of balanced blood product transfusion are most dramatic among patients likely to die as a result of bleeding, within the first 6 h of injury (9, 16–19). Delays in achieving this ratio (most often caused by processing time to thaw fresh frozen plasma) at the trauma center are associated with increased mortality (20). Moving blood-based resuscitation out of the hospital, closer to the patient was the next step.

The impact of prehospital transfusion on survival was evaluated retrospectively in a concurrent cohort study of 502 U.S. combat casualties with traumatic limb amputation or hemorrhagic shock who were evacuated by helicopter (21). Pre-hospital blood product transfusion was associated with greater survival (mortality hazard ratio 0.26 and 0.39, at 24 h and 30 days, respectively). Initiation of transfusion to treat hemorrhagic shock within 15 min of MEDEVAC rescue (median 36 min after injury) was associated with improved survival (mortality hazard ratio = 0.17), but delays beyond that eliminated the effect. More recently, Sperry et al. conducted a civilian prospective, randomized, multicenter study of 501 trauma patients at risk of hemorrhagic shock who were transported by helicopter (22). In this study, medical evacuation took place primarily from the point of injury to the trauma center and to a lesser extent (~20% of evacuated patients) after pick-up from a referral hospital en route to the trauma center. Patients received standard care en route with or without the addition of two units of thawed plasma prior to other resuscitation measures. While there was no difference in injury severity between groups, prehospital administration of plasma resulted in reduced 30 day mortality (23.2 vs. 33.0%, *p* = 0.03), compared to the standard of care group. In another prospective, randomized trial, pre-hospital plasma resuscitation was compared to saline in patients at an urban trauma center with rapid ground transport (23). No benefit of pre-hospital plasma was reported. These apparently contradictory results were explored by a *post-hoc* analysis of the combined data from these two clinical trials, which showed when pre-hospital transport time was longer than 20 min, mortality was increased among patients who received crystalloid-based resuscitation (HR 2.12; 95% CI, 1.05-4.30; *P* = 0.04), while increased mortality was not observed with >20 min transport times in patients receiving pre-hospital plasma (HR 0.78; 95% CI, 0.40-1.51; *P* = 0.46) (24). After accounting for average time from injury to arrival on scene, the findings suggested that plasma transfusion should be initiated ≤40 min from injury to improve survival. Other studies have demonstrated that pre-hospital pRBC transfusion reduces mortality, and that pre-hospital transfusion of both plasma and pRBC is better than either alone (22, 25, 26).

Evidence to inform veterinarians about the importance of early blood-based resuscitation in dogs in lacking. However, based on lessons learned from human combat casualties over the past decade, canine blood products have been pushed into far forward environments to support early hemostatic resuscitation in military working dogs (MWD). This is particularly important given the high rate of pre-hospital death for severely injured MWDs. In a recent study of 193 MWDs who sustained trauma in theater, over 90% of those who did not survive, died before they reached a veterinarian (27).

Numerous products are available for hemostatic resuscitation in the canine patient today (28). Whole blood is available from veterinary blood banks and can be acquired locally without the need for processing. pRBCs are available from commercial blood banks and can deliver concentrated oxygen carrying capacity. Plasma is available as both fresh frozen plasma and liquid (never frozen) plasma. Canine FDP is currently in development (see further information below). Platelets are typically available in three forms – fresh platelets (stored at room temperature), canine frozen platelet concentrate, and lyophilized platelets.

Other blood based products that are potentially available for hemostatic resuscitation include canine albumin which can deliver a natural colloid to expand effective circulating volume (29). While albumin products have been used experimentally for the treatment of hemorrhagic shock (30) use of hypo-oncotic, 4% albumin solution was not advantageous in treating people with traumatic brain injury (31). Definitive evidence about the utility of albumin in trauma is currently lacking. Canine cryoprecipitate is also commercially available. Cryoprecipitate has been used in management of human trauma patients primarily to provide additional fibrinogen when hypofibrinogenemia is identified in people with trauma induced coagulopathy (32), however, no evidence for use in small animals is currently available. Finally, hemoglobin based oxygen carriers have been used in veterinary medicine (33). Although not currently commercially available, these cross-linked hemoglobin products are stable at room temperature, have a long shelf life, obviate the need for typing or crossmatching and can provide short term oxygen carrying capacity in dogs (34). Because of the potential for hemoglobin based oxygen carriers to provide oxygen carrying capacity to injured MWDs in austere environments, the Department of Defense is currently funding development of these products for use in dogs. While all of these products can be considered for hemostatic resuscitation, this article will focus on whole blood which is thought to be the most efficacious for hemostatic resuscitation, freeze dried plasma which is likely to have wide application due to logistical advantages, and the use of platelet products which is a rapidly developing field.

## Chilled Whole Blood

Although the importance of blood products was recognized in combat support hospitals and forward surgery (often the first opportunity for transfusion and surgery), logistical constraints limited provision of blood products, especially platelets (35). Fresh whole blood transfusion (collected locally from volunteer service members) became a favored approach when platelets were not available. The efficacy and ease of use of whole blood were recognized, but shelf-life was initially limited to less than a day. This led to US Government programs to develop advanced, more supportable blood products (36).

Modern use of whole blood (WB) transfusion was “rediscovered” during Operations Enduring Freedom and Iraqi Freedom in an effort to meet the needs of surgeons who had no other source of platelets for forward surgery due to supply problems and short shelf-life (37). WB is advantageous because there are no RBC or platelet additive solutions, and therefore WB is less dilute with a higher concentration of coagulation factors and a higher hematocrit than can be achieved by combining component products (14, 38). The use of WB exposes the recipient to fewer donors than the equivalent amount of component products (2). The "walking blood bank,” wherein service members are called to donate in mass casualty scenarios, can provide a source of warm, fresh WB on demand, and resuscitation with this product has been associated with increased casualty survival compared to component therapy (39, 40). Warm fresh WB can be stored at room temperature for short periods, offering logistical as well as patient benefits. However, the precision required to acquire, properly screen donors, and administer the product are such that fresh WB should be used to augment, but not replace, an emergency whole blood supply comprised of chilled whole blood (CWB). Despite the need for cold storage, CWB offers the advantage of delivering all blood components to a recipient with one storage temperature (1-6°C) whereas traditional component therapy requires three: pRBC (1-6°C), FFP (≤ -20°C, plus thawing bath) and platelets (22°C). Human CWB has been shown to maintain hemostatic capacity for up to 21 days after refrigeration. Though platelet aggregometry studies reveal reduced platelet aggregation as a function of time following cold storage, their ability to form a firm clot is preserved (41). In a civilian setting, CWB used in trauma resuscitation was recently associated with a 53% reduction in blood product transfusion and a two-fold increase in survival compared to component therapy (42). Another recent study in two civilian level 1 trauma centers showed that injured patients resuscitated with CWB had higher hematocrits and decreased trauma bay mortality compared to those resuscitated with component therapy. However; there was no difference in total amounts of blood products transfused and no difference in 30-day mortality between these two groups (43). Chilled whole blood has been used to treat US combat casualties at the point of injury since 2016 and, according to US military clinical practice guidelines, is the preferred resuscitation product for pre-hospital transfusion (44).

With similar characteristics and advantages as in people, canine WB may be the ideal resuscitation fluid for dogs with hemorrhagic shock. The “walking blood bank” is familiar to many in veterinary practice as this model has served as a reliable system for obtaining fresh WB for transfusion in both general and referral practices, as well as in military settings (45). Required expertise and equipment is minimal for collection and no processing is necessary. Compared to ABO type-specific whole blood in people, canine whole blood transfusion poses less risk of hemolytic transfusion reaction in naive patients and infectious disease risks are minimal in properly screened and maintained donors (28). Guidelines for the screening of blood donors have been established and should be followed when circumstances permit (46). In emergent circumstances DEA 1 negative WB is likely optimal but it is also reasonable to consider the use of DEA 1 positive WB in transfusion naive recipients (45). Nonetheless, whole blood transfusion for small animal trauma is uncommon, representing only 11% of blood products administered (47). The inherent lag of a demand-based fresh WB donor system is a likely contributor. Prompted by advances in human CWB storage, the potential for canine CWB in trauma resuscitation has been recently explored. A recent study examined the hemostatic capacity of canine CWB stored at 4°C (48) and found it to be very similar to human CWB (41); both had loss of platelet aggregation while preserving the ability to form a strong clot through 21 days. Given this *in vitro* similarity, research is needed to determine if canine CWB has similar clinical benefits to human CWB for trauma resuscitation. The possibility of prolonged platelet function in canine CWB mitigates the obstacle of donor collection and opens the door for more dogs to receive early, balanced transfusion for severe, trauma-induced hemorrhage.

The US military's clinical practice guidelines currently recommend canine whole blood as the first choice for resuscitation of MWDs in hemorrhagic shock, to be administered as soon as feasible after injury and when conditions are safe for casualty care (45, 49). Deployed military veterinarians have effectively provided lifesaving transfusions to MWDs in austere settings in both Iraq and Afghanistan (27). The authors are aware of a case where a MWD suffered a gunshot wound to the thorax with significant hemorrhage and survived after treatment and early resuscitation with a hemoglobin based oxygen carrier during MEDEVAC followed by canine WB at the next level of care. A pilot program has been successfully implemented by the US military to ship and maintain commercial canine CWB from the United States through the medical transport system into forward locations. (Edwards personal communication). Canine WB is now being staged as close as possible to potential points of injury and this strategy has proven successful in the field.

## Freeze Dried Plasma

In the most austere settings where an unbroken cold chain is not possible, balanced transfusion may not be achievable. In these cases, an environmentally stable “bridge,” such as FDP, is needed to temporize the casualty until full blood-based resuscitation can occur. FDP was first developed in the 1930's as a pooled and lyophilized product that was sterilely packaged, stored at ambient temperature, and reconstituted immediately prior to administration. Large-scale production and utilization of FDP occurred in World War II where it was principally used as a bridge to full resuscitation with WB. After over 6.5 million units had been used by British and US troops (3), it became apparent that plasma transfusion was associated with hepatitis transmission (50). Although effective in resuscitation, FDP was largely abandoned by most countries by the mid-1960s. The French military continued to utilize FDP in the Indochina war but its production was halted in 1985 due to concerns about HIV transmission, but later restarted (51).

Modern pathogen detection and reduction technology has now made it possible to safely produce FDP. Currently there are three human FDP products that are commercially available (South African, French, and German products) and have been reviewed elsewhere (3, 51). Several US companies are attempting to gain FDA approval for their FDP products in the US (52). At the time of this writing, the U.S. Department of Defense has been granted an Emergency Use Authorization by the FDA to utilize the French FDP product to resuscitate American troops in combat zones.

A great deal of research has been conducted within the last few years demonstrating the feasibility and efficacy of FDP. In a rodent model of hemorrhagic shock, FDP was equivalent to FFP in mitigating endotheliopathy and in decreasing pulmonary vascular permeability, edema and inflammation, compared to crystalloids (53). In a swine polytrauma model, resuscitation with FDP improved survival compared to resuscitation with hydroxyethyl starch (54). In a retrospective civilian trauma study, people who received FDP had fewer massive transfusions, and more quickly achieved a 1:1 pRBC:plasma ratio compared to those who received FFP, and FDP was ready for administration much more rapidly than FFP (15 min compared to 95 min) (55). When administered to trauma patients transported by helicopter emergency medical services in the United Kingdom, pre-hospital FDP reduced the need for RBC transfusion by 18% and prevented a 71 min delay to plasma administration (56). In studies published by the Israeli Defense Force examining FDP use in casualties, investigators found that FDP was logistically feasible in a pre-hospital setting and was associated with more favorable patient coagulation profiles compared to those who did not receive FDP, in both pediatric and adult patients (57, 58).

There is some historical data on the use of canine FDP (cFDP) in veterinary medicine. In 1962, a study documented the administration of cFDP to dogs using a product manufactured by Pitman-Moore, Inc. For the initial study, 10 normal dogs were administered cFDP subcutaneously followed by an IV infusion of cFDP. The author noted that 3 out of 10 dogs had a mild reaction to the cFDP. Subsequently the author administered cFDP to 47 dogs in shock and concluded that the cFDP was “usually effective” (although the author did not elaborate further) (59). Recently, the Department of Defense has funded two companies to develop a cFDP. At the time of this writing, cFDP is close to meeting the regulatory requirements needed for commercialization. (Edwards personal communication) Pilot data were recently presented documenting initial safety and efficacy of one cFDP product. In this study, six dogs underwent a therapeutic plasma exchange, with one group receiving cFFP and the other receiving cFDP. The study reported no adverse events in any of the dogs that received cFDP and perfusion and coagulation parameters that were within reference ranges and similar between groups (60). Another *in vitro* study showed that the two cFDP developmental products were similar to canine FFP in coagulation factor and thromboelastographic parameters, and retained acceptable hemostatic function for up to 14 days refrigerated after reconstitution (61). The logistic benefits of cFDP (>1 year shelf life, ambient storage temperature, and rapid reconstitution) offer the potential to begin hemostatic resuscitation of severely injured dogs as soon as possible after injury. In military practice, this is in the field when conditions are safe. In civilian practice, this may be at first-stop emergency or primary care facilities where conventional blood products may not be available. Using plasma as a bridge to definitive hemorrhage control and complete blood-based resuscitation increases survival in people (22); similarly, administration of cFDP prior to transfer to a blood-equipped facility could potentially improve outcomes in dogs with severe traumatic hemorrhage. Currently manufacturers of cFDP recommend the products be reconstituted into an isotonic product but research is needed to determine if a hypertonic FDP could be more efficacious in resuscitation of severely injured dogs.

Canine plasma products are currently recommended for MWDs in hemorrhagic shock after severe trauma (45, 49) as a bridge to full resuscitation with either whole blood or balanced ratios of plasma:pRBCs, and have been used effectively as a resuscitation fluid during recent conflicts. At the time of this writing, the US Army is using one cFDP product as part of a veterinary clinical study. This cFDP or thawed canine FFP is often carried by certain combat medics and handlers on high risk missions so it is readily available for early hemostatic resuscitation in MWDs that may suffer trauma in a far forward austere environment.

## Chilled Platelets

Platelet administration plays an integral role in balanced transfusion and, in people, may improve survival from trauma (11, 13, 62). Both retrospective and prospective data suggest that lower platelet count on admission is associated with increased risk of death among human trauma patients (63, 64). Platelets may become hypofunctional following injury and data suggest that an early platelet transfusion strategy has utility beyond simply replacing what is lost. A prospective study of 101 critically injured human trauma patients found that platelet hypofunction carries a 10-fold increase in mortality (65). Platelet hypofunction following trauma has also been demonstrated in rodent and pig models (66, 67). Therefore, optimization of hemostatic resuscitation in the trauma patient requires consideration of both platelet number and function.

Traditionally, platelets are stored at room temperature and are most often administered to prevent bleeding in patients with hematologic malignancies (68). The rationale for room temperature storage of platelets is based on increased circulation times for platelets stored at 22°C compared to chilled platelets (69). Due to concern for bacterial contamination, platelets can only be stored at room temperature for 5-7 days under constant agitation, and older units of platelets carry with them an increased risk of adverse events in people (70). Additionally, older platelet units develop a storage lesion that renders them less hemostatically effective (71–73). The short storage life and need for constant agitation renders traditional platelet products challenging to use in operational environments; thus, the US Department of Defense has expended considerable resources to rediscover the benefits of cold stored platelets (CSP) (74).

Cold storage of platelets is not new. Platelets were commonly stored at 1-6°C until the 1970s when the increased circulation time of room temperature platelets was recognized and thought to be highly important (75). While this may be an important characteristic of platelets transfused for prevention of bleeding, platelets transfused for acute traumatic hemorrhage are required to function immediately upon administration, but do not require a lengthy circulation time. Chilled platelets can be stored for up to 2 weeks. *In vitro* studies have recently shown that aggregation response, clot kinetics and firmness, and adhesion to collagen were all better preserved in CSP compared to room temperature platelets (72, 74). Chilling platelets reduces mitochondrial activity, reactive oxygen species production and inflammatory mediators, thereby extending their viability (76, 77). Wu and colleagues showed that cold platelets (transfused as CWB) were equivalently incorporated into clots as those of fresh WB in a rat model of acute traumatic coagulopathy (78). Similarly, Torres and colleagues demonstrated in a rat hemorrhage model that CSP participated equally in clot formation when compared to room temperature platelets (79). A randomized clinical trial in Norway demonstrated that 7 day CSP transfusion is at least equally as effective as room temperature platelets in treating post-operative bleeding after cardiac surgery in people (80). When paired with the logistical superiority of prolonged storage life and decreased risk of bacterial contamination, the functional equivalence of CSP prompted the military to request an FDA variance allowing 14 day cold storage of platelets. This variance, now granted, enables the military to transport CSP from the US into austere environments for use in wounded service members. In February 2020, a South Texas civilian blood bank was also granted a license for 14 day storage of CSP and it is expected that the product will be more widely authorized throughout the US in the coming years.

With the exception of one study examining the *in vitro* hemostatic capacity of canine chilled whole blood (48), the understanding of canine CSP is adapted from other species. However, the potential for an extended storage life may help offset some of the challenges of platelet concentrate preparation and transport, as well as reduce product wastage given the high cost (81). Veterinary research is needed to assess whether platelets play a vital role in hemostatic resuscitation as shown in people. If administration of platelets early after injury is as important in small animals as it is in humans, CSP may find a place in veterinary centers with a high enough trauma load to justify the product. Apart from those contained in whole blood, CSP (and platelet transfusion in general) is not addressed in the current MWD clinical practice guidelines. In part, this is because canine platelet collection (*via* apheresis) is not yet practical in the deployed setting and shipment of US manufactured canine platelet concentrate for cold storage is likely to result in product wastage given the preference for whole blood resuscitation. A lyophilized platelet product which is better suited for the tactical environment than CSP is currently available commercially for dogs, but not humans. Lyophilized platelet products have similar clinical performance to other platelet preparations for treatment of thrombocytopenic bleeding (82, 83). A Department of Defense funded multicenter randomized clinical trial evaluating canine lyophilized products (plasma and platelets) for early canine trauma resuscitation is underway. Comparisons between CSP and lyophilized platelets for treatment of canine hemorrhage have not been performed.

## Implementation and Potential Challenges in Veterinary Medicine

The U.S. military's ability to rapidly field novel products and strategies has greatly advanced trauma medicine and has undoubtedly saved both military and civilian lives. In fact, the U.S. military is the primary funding source for trauma research in America. Veterinary transfusion medicine has also progressed substantially in the last 20 years. Trauma resuscitation guidelines for MWD have mimicked those of human combat casualties. Standard blood products are widely available at civilian referral institutions, with a recent survey-based study citing that 100 and >90% of referral centers maintain supplies of pRBC and FFP, respectively, (84). Less frequently, these practices carry cryoprecipitate, liquid plasma, stored plasma, lyophilized platelet products, and WB. Fresh WB is sometimes available on demand through volunteer or in-house donor programs, and in rare cases platelet units can be manufactured at blood banks associated with large veterinary hospitals. A massive transfusion study in dogs (defined as administration of 50% blood volume in 3 h or 100% in 24 h) found that both pRBC and FFP were administered, but that none of the 15 dogs received WB (85). In a more recent study of trauma transfusion practices in dogs, 5 of 45 dogs received WB and 10 dogs underwent massive transfusion (47). Clearly, advanced trauma transfusion in canine patients is feasible and has the potential to be beneficial. However, financial considerations often limit what services, technologies and therapies can be provided to canine patients in the civilian sector and advances such as those described above may increase cost to clients.

Modern human trauma systems, both military and civilian, incorporate on-scene and en route care by trained personnel, and rapid transport to trauma centers for advanced care, which is life-saving (86). Veterinary medicine does not have a similar evacuation system in place for injured animals and care en route for veterinary patients is rarely practiced. Veterinary trauma patients are often quickly transported by their owners seeking veterinary care and are commonly presented to the closest facility which may or may not be optimized for trauma resuscitation. Though efforts are made in earnest to stabilize, many severely injured animals require transfer to emergency and referral facilities, potentially requiring prolonged transport times. Even with planned transfer, the importance of what has been learned about early blood-based resuscitation in the pre-hospital environment should be considered and veterinary practices may wish to apply these strategies in various practice settings when feasible. To this end, recent military advances have shifted the paradigm of transfusion medicine, making blood products more available and more durable in the minimally equipped, austere environment. This may pave a way for veterinary practices, especially those that are geographically isolated or resource constrained, to participate more extensively in early hemostatic resuscitation for severely injured patients. With the (re)introduction of CWB, FDP, and CSP, the availability of a lyophilized platelet product, and relatively low costs for associated equipment, it may become possible for smaller practices to stock blood products with reasonable shelf-lives, reducing the risk of product wastage. This may enable veterinary practitioners to offer life-saving hemostatic resuscitation when a severely injured trauma patient is presented for care. Although financial limitations are likely to remain an obstacle to full implementation of the lessons learned on the battlefield, awareness and understanding of these trauma advancements can help the veterinary practitioner offer a range of care options to suit the needs of all clients. Additional robust clinical and preclinical studies in target species will be necessary to guide best practices and improve trauma outcomes in veterinary patients. In particular, research is needed to determine which canine patients (based on severity of injuries, degree of shock, etc.) would benefit from hemostatic resuscitation over crystalloid based resuscitation strategies. Nevertheless, the abundance of evidence in other species strongly suggests that patients suffering from severe trauma significantly benefit from hemostatic resuscitation and many of these practices can be utilized in veterinary patients today.

## Author Contributions

TE and AP obtained funding for the manuscript. All authors participated in the drafting of the manuscript. All authors reviewed and edited the manuscript.

## Conflict of Interest

EM is affiliated with Bodevet Inc. who has developed canine lyophilized plasma and lyophilized platelets. The remaining authors declare that the research was conducted in the absence of any commercial or financial relationships that could be construed as a potential conflict of interest.
